# Physical Outdoor Activity versus Indoor Activity: Their Influence on Environmental Behaviors

**DOI:** 10.3390/ijerph14070797

**Published:** 2017-07-17

**Authors:** Wei-Ta Fang, Eric Ng, Mei-Chuan Chang

**Affiliations:** 1 Graduate Institute of Environmental Education, National Taiwan Normal University, Taipei 116, Taiwan; db1305@yahoo.com.tw; 2 School of Management and Enterprise, University of Southern Queensland, Toowoomba, QLD 4350, Australia

**Keywords:** environmental behaviors, physical outdoor activity, primary school children, social norms

## Abstract

There are strong evidences linking physical outdoor activity and health benefits; however, little is known about the impact on environmental behaviors. Thus, this study aims to close this gap by investigating the influence of physical outdoor activity on environmental behaviors. A total of 416 surveys were distributed to students in eight public primary schools located near the Hsinchu Science and Industrial Park in Taiwan. Findings from the analysis revealed that subjective norms had a more influential effect on environmental behaviors for participants who engaged in physical activity at outdoor parks. In contrast, descriptive norms had a direct predictive impact on environmental behaviors for participants whose main physical activity venue was at the indoor after-school centers. Research results also highlighted attitude as the strongest predictive variable influence on environmental behaviors for children who engaged in physical indoor and outdoor activities.

## 1. Introduction

During the period of human development, experiences gained through interactions with the environment can have a significant impact on a person’s perception of the environment [1]. In particular, children are known to have more positive experiences in natural outdoor environments than adults since they have not yet been accustomed to the unnatural environments. Studies also revealed that children’s development towards the values of nature needs to be supported through regular personal participation and interaction with a diverse natural setting, and this can have significant effects on their awareness and behaviors for environmentally friendly initiatives [2,3,4]. Through this, pro-environmental behavior can be further developed where children will be more conscious in seeking to minimize the negative impact of their actions on nature.

Nowadays, children have fewer opportunities for outdoor play and this can be attributed to the increasing children’s safety concern by parents, and working families not able to supervise their children after school, therefore leaving them to stay indoors or attend supervised after-school activities [5]. At a macro level, natural outdoor activity venues for children have also become increasingly scarce with rapid urban development. This is evident with the rapid economic development and industrial construction in many emerging industrialized countries, particularly in Asia where production hubs have been established in recent years. As a result, there are limited outdoor activity venues for children living in urban areas, which minimizes their physical outdoor activity and subsequently affects their interactions with the natural environment. This phenomenon has also contributed to physical inactivity, which is a growing concern to many countries and has been identified as the fourth leading risk factor for global mortality [6]. In the context of outdoor activity venues in urban areas of Taiwan, these were relatively small areas with high ratios of impervious pavement and a scarcity of animals and plants. Children generally had very limited time (i.e., nearly 50% of the children in Taiwan visit daycare centers and intense school routines) to engage in physical outdoor activities. As a result of such space and time constraints, indoor after-school centers had become a principal venue for children to participate in physical activities.

Accordingly, physical activity can be defined as any body movement produced by skeletal muscles that results in energy expenditure [7]. There are evidences supporting a close correlation between health and physical activity, and the exposure to nature may have direct health benefits [6,8,9]. Other studies have also investigated several different aspects of physical activity, such as the types of physical activity [10], measurement of physical activity [11], social and environmental influences on physical activity [12,13,14], promoting physical activity [15,16], and physical activity guidelines [17,18]. However, very few studies have attempted to bring the two fields of physical activity and environmental behaviors together, and explore their relationship. Therefore, the key focus of this study is to fill this gap by investigating specifically the influence of children’s physical outdoor activity on environmental behaviors. 

Researchers have used the Social Attribution Theory to explain the causes of human behavior. This theory posits that a behavior can be influenced by situations that arise from external social factors or internal reasoning through personal attitudes [19,20,21]. Norms are social factors beyond attitudes that shape people’s behaviors [22] and can be classified as (1) subjective norms, and (2) descriptive norms. Subjective norms are regarded as “the perceived social pressure to perform or not to perform the behavior” in question or in a specific situation, whereby the greater the pressure to support or oppose a norm, the stronger the effect of behaviors [23]. On the other hand, descriptive norms represent individuals’ perceived practices of most people with whom one has interacted in the same space, and its impact on human behaviors often seems unconscious [24]. This study has adapted the Social Attribution Theory to examine the influence of the children’s physical indoor and outdoor activity on environmental attitude and behaviors, and how their social norms (subjective norms or descriptive norms) have a direct/indirect association with their environmental behaviors.

## 2. Materials and Methods

### 2.1. Research Area

This study was conducted in Hsinchu, Taiwan, a major industrial city in Asia. Specifically, the research was undertaken at the Hsinchu Science Park, which was founded in 1980 with an area of 653 hectares developed at the Hsinchu Park [25]. During 2015, there were 478 companies in the Hsinchu Science Park with a total of 149,100 employees that generated an overall revenue of approximately NT$1.1 trillion [25]. There were also several schools situated in the Hsinchu Science Park area that cater to the needs of many families working there.

### 2.2. Participants

The adoption of the probability cluster sampling method in this study resulted in the selection of eight public elementary schools around the Hsinchu Science Park. Two classes (one each from grades five and six with approximately 60 children in total) were randomly selected from each of these eight elementary schools. The participants involved in this study were children (from grades five to six, ages 11 to 13) and were stratified by gender, and two purposefully selected physical activity venues (i.e., indoor after-school centers and outdoor parks). The rationale for selecting this specific sample of grades five and six children was primarily because they were at the early adolescence stage (generally ages 11 to 14), which was considered a critical period to shaping their environmental cognition, affective values, environmental behavior, and physical activity behavior [26,27,28]. Furthermore, children in this age group were deemed to have the ability to better understand and therefore adequately answer the questionnaire. To determine if participants were eligible to participate in this study, a screening question was set at the beginning of the survey. Participants must have indicated indoor after-school centers or outdoor parks as their main physical activity venue to qualify and subsequently complete the questionnaire.

Written consent forms were sent to parents, children, class teachers and school principals to obtain their agreement to participate in this research study, and at the same time informing them of their rights to withdraw at any time. The questionnaire survey had proceeded after permissions to participate had all been received and was conducted in the classroom setting at the respective schools. The participants were given approximately 15 min in class to complete the questionnaire and the class teacher collated them when completed. The National Taiwan Normal University Research Ethics Committee concluded that our study does not fall within the scope of the Human Subjects Research Act. They approved the study protocol (201707HS001) and agreed with active informed consent by the class teachers and school principals with parents having the option to opt their child out of the study. A total of 416 questionnaires were distributed and were all returned. However, 31 were considered invalid because they failed to indicate either indoor after-school centers or outdoor parks as their main physical activity venue in the screening question that was set at the beginning of the survey, with the remaining 385 being analyzed.

### 2.3. Measures

This study was based on a substantial body of literature demonstrating the cognitive and psychological influence on environmental behavior research (e.g., [29,30,31]). Therefore, this study specifically sought to measure two key dimensions: (1) attitude, and (2) social norms (including subjective and descriptive norms) that were considered influential to environmental behaviors [32,33,34].

This research used the questionnaire survey method, which was comprised of background and psychological variables developed in align with the two key dimensions identified earlier. The questions in the questionnaire were mainly adapted from previously conducted environmental behavior research studies, specifically related to the attitude [35], and subjective and descriptive norms [36,37] dimensions. The questionnaire survey was pre-tested with three experts and 150 children between grades five and six, to determine the appropriateness and understanding of the questions. As a result, some minor changes were made to the questionnaire and subsequently used in the conduct of the actual survey. These minor changes mainly involved only simplifying the wordings to include more colloquial and clear terms for children to understand in their local context, but retained the original meaning of the items adapted from the earlier mentioned studies. Some examples of these changes include: “I believe that most of my acquaintances expect that I join the environmental club” to “People I know are supportive of my participation in environmental protection clubs”; “I believe that most of my acquaintances join environmental club” to “People I know join environmental protection clubs”; “I would be willing to take part in outdoor environmental activities” to “I participate in environmental activities conducted outdoors”.

This research utilized the Statistical Package for the Social Sciences (SPSS) software program to conduct the analysis. Frequency analysis was used to determine the total number of occurrences, the mean and standard deviation (SD) scores for the demographic questions and items in the key dimensions (i.e., attitude, subjective norms, descriptive norms, and environmental behaviors). The Pearson Correlation technique was used to measure the strength and direction of relationship that exists between these key dimensions. The multiple regression analysis was used to predict the influence of attitude, subjective and descriptive norms on environmental behaviors. A five-point Likert scale (i.e., 1 = “Strongly disagree” to 5 = “Strongly agree”) was adopted for the measurement. The Cronbach’s α values of the respective dimensions were attitude (0.835), subjective norms (0.871), descriptive norms (0.821), and environmental behaviors (0.858), which demonstrated internal reliability since their values were greater than the required 0.7. In addition, the Kaiser–Meyer–Olkin value was recorded at 0.909 (which was greater than the required value of 0.8), and the Spherical Bartlett test value was noted at 1915.172, *p* < 0.001. Factor and principal component analyses were subsequently conducted and the factor loading of each question in the three dimensions has exceeded the value of 0.4. Therefore, the measured psychological variable scales were considered reliable.

## 3. Results

### 3.1. Descriptive Statistics

Findings indicated that both males (49.9%) and females (50.1%) were equally represented in this study, with 191 grade five students and 194 grade six students. The two main physical activity venues investigated were indoor after-school centers and outdoor parks, which accounted for 54.5% and 45.5%, respectively. Results revealed significant differences between females and males in terms of their attitude (df = 383, two-tailed, *t* = 3.100 > 1.967, *p* = 0.002) and environmental behaviors (df = 383, two-tailed, *t* = 2.379 > 1.967, *p* = 0.018). There was also a significant difference between students whose main physical activity venue was at indoor after-school centers and outdoor parks in relation to their environmental behaviors (df = 383, two-tailed, *t* = 2.246 > 1.967, *p* = 0.025). Table 1 below briefly outlines selected demographic findings regarding subjective norm, descriptive norm, attitude, and environmental behaviors. 

Findings for the attitude-related items are presented in Table 2. Among the five attitude-related items, participation in environmental protection clubs received the highest mean score, and this was followed by being an environmental volunteer; participation and enjoyment in beach cleaning activities; participation in environmental activities and join clubs; and the infrequent usage of air conditioning and lesser consumption of chilled beverages. The results indicated an internal consistency reliability measurement with the Cronbach’s α value of 0.835 for the attitude-related items.

There were five items (water conservation, going outdoor and reduced usage of air conditioning, waste sorting, self-prepared water cup and cutlery, and participation in environmental protection clubs) associated with subjective norms and Table 3 presents their respective mean scores. There was a consistent reliable measurement for the subjective norms-related items with the Cronbach’s α value of 0.871.

Findings (see Table 4) also indicated that the two descriptive norms-related items; not littering arbitrarily, and joining environmental protection clubs had the highest and lowest mean score, respectively. Other items include: awareness of water conservation; self-prepared cutlery; and going outdoor instead of staying indoor to reduce usage of air conditioning. The Cronbach’s α value of 0.821 suggested internal consistency for the descriptive norms-related items.

Results (see Table 5) revealed that there were five items about the impact of social norms on environmental behaviors, and of which persuade others to sort waste had the highest mean score. This was followed by going outdoor instead of staying indoor watching television programmes and using a computer; participation in outdoor environmental activities; participation in conducting surveys on animal and plant-related activities in the vicinity of the community; and visiting parks and volunteering. The internal consistency reliability for the environmental behaviors-related items was measured with the Cronbach’s α value of 0.850. 

### 3.2. Correlation Analysis

As shown in Table 6, results of the correlation analysis revealed moderate correlation between attitude and subjective norms. The correlation between attitude and environmental behaviors remained the highest, indicating a high level of correlation; the correlation coefficient of subjective norms and environmental behaviors, and between descriptive norms and environmental behaviors, both suggesting a moderate correlation. Therefore, social norms, attitude, and environmental behaviors are correlated (e.g., [37,38]).

### 3.3. Regression and Path Analysis

**Figure 1:**
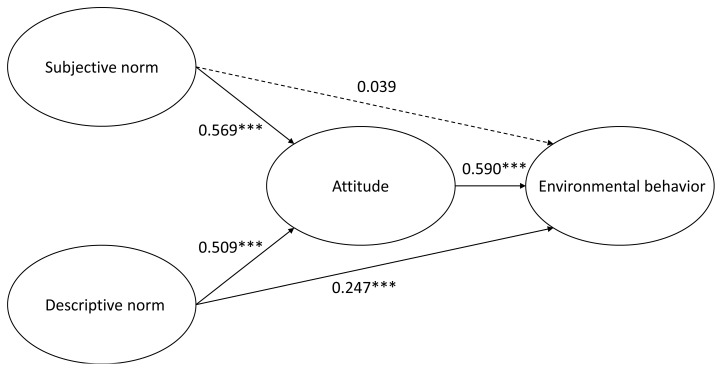
Path diagram on the social norms and environmental behaviors of children whose physical activity venue was at the indoor after-school centers.

Results of the regression analysis revealed differing social norms that affect environmental behaviors at the indoor after-school centers and outdoor parks. As shown in Table 7, descriptive norms had a direct predictive influence on environmental behaviors for those children whose physical activity venue was at the indoor after-school centers. The influence of subjective norms on environmental behaviors was insignificant. In addition, Figure 1 also displays the path analysis on the relationship between social norms and environmental behaviors of children whose physical activity venue was at the indoor after-school centers. According to the path analysis, descriptive norms had a positive direct effect on environmental behaviors when physical activities occurred at the indoor after-school centers. On the contrary, subjective norms did not result in a direct path to environmental behaviors, but instead could be developed through environmentally friendly attitudes.

**Figure 2:**
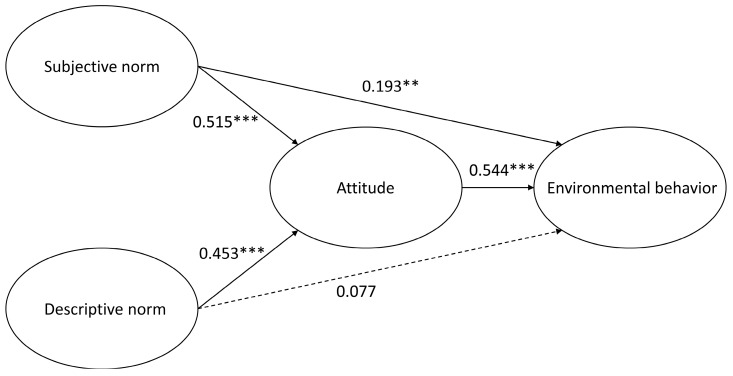
Path diagram on the social norms and environmental behaviors of children whose physical activity venue was at the outdoor parks.

In contrast, where children’s physical activity venue was at the outdoor parks, subjective norms had a direct predictive impact on environmental behaviors, whereas the influence of descriptive norms was insignificant (please refer to Table 8). As shown in Figure 2, the path analysis presented the relationship between social norms and environmental behaviors of children whose physical activity venue was at the outdoor parks. The path analysis revealed that children who engaged in physical activities at the outdoor parks were affected by their subjective norms (e.g., own environmental awareness) and had a positive direct impact on environmental behaviors. However, there was no direct path established between descriptive norms with environmental behaviors, but such a relationship could be developed through environmentally friendly attitudes.

## 4. Discussion

### 4.1. Influence of Attitudes

Based on the Social Attribution Theory framework as outlined earlier, this study investigated the influence of the children’s physical indoor and outdoor activity on environmental attitude and behaviors, and how their social norms (subjective norms or descriptive norms) affect their environmental behaviors. The research results revealed that attitude was the predictive variable with the strongest influence on environmental behaviors for children who engaged in physical indoor and outdoor activities. Social norms also affected attitude, which subsequently impacted on environmental behaviors. 

Previous studies conducted using the Social Attribution Theory had adopted a more simplistic model that tested social norms (without differentiating descriptive and subjective norms) in terms of situational attribution based on external social factors, and examined behavioral intentions (but not behavior) as the dependent variable. This study has attempted to fill the gap and further refined the Social Attribution Theory by testing the descriptive and subjective norms separately to determine their specific path and level of influence on environmental behaviors. Specifically in this study, for physical activities at the indoor after-school centers, results highlighted not only the indirect path through which descriptive norms affect attitude, which had a flow on effect on environmental behaviors, but also a direct positive path that affects environmental behaviors. However, subjective norms did not result in a direct path to environmental behaviors, but instead could be indirectly developed through attitude. On the other hand, for physical activities at the outdoor parks, findings indicated that subjective norms had a positive direct impact on environmental behaviors, as well as an indirect path affecting environmental behaviors through attitude, while descriptive norms had no direct path affecting environmental behaviors, but an indirect influence could be established through attitude. Therefore, the research findings supported the previous studies conducted (e.g., [39,40]), and extended the understanding of the Social Attribution Theory through the identification of the specific path and influence of descriptive and subjective norms on environmental behaviors.

### 4.2. Influence of Social Norms (Subjective and Descriptive Norms)

Findings revealed that subjective norms played a critical role affecting children’s environmentally friendly behavior when they engaged in physical activities at the outdoor parks. This could be explained by a lack of supervision (e.g., by the park management) on the children’s environmentally friendly behaviors, and such an open environment had no strict tracking mechanism of such behaviors when compared with the indoor after-school centers. Behaviors generated by subjective norms were considered semiautonomous acts, and although introspection and reflection which are governed by strong personal ethics were absent, subjective norms were not submissive acts. Instead, subjective norms emerged when an individual placed oneself in the shoes of the others (e.g., relatives, friends) and as a result displaying appropriate behaviors. Furthermore, when children were at the outdoor parks, they tend to be more caring about the natural environment and form a psychological bond with nature, which could contribute to their environmentally friendly behaviors [2,41]. Therefore, subjective norms known to the children became essential and could positively influence their environmentally friendly behaviors.

In contrast to subjective norms, descriptive norms were based on behavioral criteria that had clear requirements and expectations to follow. The results indicated that descriptive norms had minimal influence on children who often engaged in physical activities at the outdoor parks, but instead was more influential at indoor after-school centers. This could be explained by the traditional Asian cultures rooted in Taiwan where children were expected to follow the instructions given by the teachers or adults. For example, teachers would continuously monitor and ensure that the children display behaviors that conform to normative requirements at the indoor after-school centers. In such an environment of strong collectivism, children were afraid of drawing the teacher’s attention and being regarded as “different” or “abnormal”. Therefore, to avoid being in the “spotlight”, the safest way was to act the way other children behave. Such a consistent behavior of children generated group descriptive norms, which could have subsequently affected their environmental behaviors [42,43].

In summary, the research results suggest that descriptive norms were more likely to predict environmentally friendly behaviors when children engaged in physical activities at the indoor after-school centers, whereas subjective norms were regarded as more influential on children’s environmentally friendly behaviors when their primary physical activity venue was at the outdoor parks.

### 4.3. Implications, Limitations and Future Research

The findings of this research add new insights to the literature about the influence of children’s physical outdoor activity on environmental behavior. In addition, the results have implications on governmental policies, such as when making urban planning and design-related policies and decisions, due consideration is needed to retain more physical outdoor activity venues and spaces (e.g., parks) that will allow children to maximize their physical outdoor activity and maintain a healthy lifestyle.

This study investigated the influence of physical outdoor activity on environmental behaviors within the context of the primary schools located near the Hsinchu Science and Industrial Park in Taiwan, and therefore constrain the applicability of the findings to other parts of the country and sectors. A more representative sampling population should be sought and tested, in order to generalize the findings. Further research would be needed to provide comparisons between other countries to ascertain the similarities or differences in such a context. In addition, future research could also seek to investigate if parents and teachers play a critical role influencing the choice of children’s physical activity venues, which might have a subsequent effect on their environmentally friendly behaviors. An opportunity for participants to sign up for a future environmental activity could also present a view beyond self-reported environmental behaviors.

## 5. Conclusions

In conclusion, this research investigated the influence of physical indoor and outdoor activity on environmental behaviors. Findings indicated that social norms influenced children’s environmentally friendly behavior, and generated an indirect path that affected attitude, which subsequently changed environmental behaviors through a direct path. Social norms also had a direct effect on environmental behaviors.

The degree of influence and path of subjective and descriptive norms varied between physical indoor and outdoor activity venues. The research results suggested that descriptive norms were critical factors affecting environmental behaviors at indoor after-school centers. In contrast, subjective norms were the major factors affecting environmental behaviors at outdoor parks where strong control or strict monitoring mechanisms were absent. 

## Figures and Tables

**Table 1:** Descriptive statistics related to the demographic questions for subjective norm, descriptive norm, attitude, and environmental behaviors items.

Variables	Frequency	Percent	Subjective Norm				Descriptive Norm				Attitude				Environmental Behaviors			
			Mean	SD	*t*	*p*	Mean	SD	*t*	*p*	Mean	SD	*t*	*p*	Mean	SD	*t*	*p*
Gender																		
Female	193	50.1	3.65	0.802	1.317	0.188	3.53	0.83	0.885	0.377	3.48	0.82	3.100	0.002	3.44	0.89	2.379	0.018
Male	192	49.9	3.53	0.927			3.46	0.86			3.22	0.86			3.22	0.92		
Grade level																		
Grade 6	194	50.4	3.59	0.83	−0.021	0.983	3.45	0.82	−1.125	0.261	3.27	0.81	−1.956	0.051	3.29	0.85	−0.804	0.422
Grade 5	191	49.6	3.59	0.90			3.54	0.87			3.43	0.88			3.37	0.97		
Main physical activity venue																		
Outdoor parks	175	45.5	3.67	0.84	1.748	0.081	3.52	0.87	0.564	0.573	3.40	0.84	0.967	0.334	3.44	0.90	2.246	0.025
Indoor after-school centers	210	54.5	3.52	0.89			3.47	0.83			3.31	0.86			3.24	0.92		

**Table 2:** Descriptive statistics for attitude-related items.

Attitude	Mean	SD
I believe participation in environmental protection clubs is useful.	3.61	1.08
I should become an environmental volunteer.	3.41	1.16
I find participation in beach cleaning activities enjoyable.	3.41	1.11
I should participate in environmental activities and join clubs.	3.30	1.07
I should use less air conditioning and drink chilled beverages less often.	3.02	1.06
Overall attitude	3.35	0.85

**Table 3:** Descriptive statistics for subjective norms-related items.

Subjective Norms	Mean	SD
People I know want me to save water.	3.71	1.05
People I know want me to go outdoors and use less air conditioning.	3.69	1.10
People I know are supportive of my waste sorting.	3.65	1.08
People I know want me to carry a water cup and cutlery when I go out.	3.63	1.04
People I know are supportive of my participation in environmental protection clubs.	3.25	1.08
Overall subjective norms	3.59	0.87

**Table 4:** Descriptive statistics for descriptive norms-related items.

Descriptive Norms	Mean	SD
People I know do not litter arbitrarily.	3.75	1.05
People I know are well aware of how to save water.	3.73	1.03
People I know carry their own cutlery when they go out.	3.53	1.07
People I know go outdoors instead of staying indoors with air conditioning.	3.28	1.21
People I know join environmental protection clubs.	3.19	1.18
Overall descriptive norms	3.50	0.85

**Table 5:** Descriptive statistics for environmental behaviors items.

Environmental Behaviors	Mean	SD
I persuade others to sort waste.	3.63	1.121
I go outdoors in my free time, rather than watching TV and sitting in front of the computer.	3.52	1.177
I participate in environmental activities conducted outdoors.	3.39	1.150
I participate in conducting surveys on animal and plant-related activities in the vicinity of the community.	3.11	1.170
I visit the park and volunteer.	3.00	1.143
Overall environmental behaviors	3.33	0.911

**Table 6:** Overall Pearson’s Product–Moment Correlation (Mean).

	Attitude	Subjective Norms	Descriptive Norms	Environmental Behaviors
Attitude	1.000			
Subjective norms	0.599	1.000		
Descriptive norms	0.515	0.661	1.000	
Environmental behaviors	0.684	0.532	0.504	1.000

All correlations are significant, *p* < 0.01 (two-tailed test).

**Table 7:** Multiple regression analysis of attitude and social norms that predict environmental behaviors of children whose physical activity venue was at the indoor after-school centers (*n* = 210).

Dependent Variable: Environmental Behaviors	First Stage of Standardized Coefficient	Second Stage of Standardized Coefficient	Third Stage of Standardized Coefficient
Attitude	0.716 ***	0.590 ***	0.573 ***
Descriptive norms		0.247 ***	0.231 ***
Subjective norms			0.039
R^2^	0.513	0.558	0.559
ΔR^2^	0.511	0.554	0.553
F(1208)	219.205 ***	130.928 ***	87.089 ***

*** *p* < 0.001.

**Table 8:** Multiple regression analysis of attitude and social norms that predict environmental behaviors of children whose physical activity venue was at the outdoor parks (*n* = 175).

Dependent Variable: Environmental Behaviors	First Stage of Standardized Coefficient	Second Stage of Standardized Coefficient	Third Stage of Standardized Coefficient
Attitude	0.644 ***	0.544 ***	0.526 ***
Subjective norms		0.193 ***	0.150
Descriptive norms			0.077
R^2^	0.414	00.441	0.444
ΔR^2^	0.411	0.435	0.435
F(1173)	122.277 ***	67.972 ***	45.595 ***

*** *p* < 0.001.
